# Belt–pulley interaction: role of the action line of friction forces

**DOI:** 10.1007/s00707-020-02724-5

**Published:** 2020-06-27

**Authors:** Evgenii Oborin

**Affiliations:** Institute of Technical Mechanics, Johannes Kepler University Linz, Altenbergerstr. 69, 4040 Linz, Austria

## Abstract

A simplified model of the belt motion with small strains is proposed. The main purpose of the modeling is to show the effects arising when the line of action of friction forces is shifted to the belt’s middle axis. The prestressed shearable model of the belt is used in this study. The differential equations of the steady state motion are integrated and combined together with the boundary conditions into two nonlinear systems of algebraic equations corresponding to the different cases of the belt behavior: presence and absence of a sliding segment. The nonlinearity results from the fact that the boundaries of the contact segments are a priori unknown. The case without sliding requires introduction of a concentrated force at the point where the belt leaves the pulley. Considerable effects of the assumptions of contact characterization on the simulation results are demonstrated.

## Introduction

In this paper, we investigate the stationary motion of an initially straight belt in contact with a rigid pulley (see Fig. 2.1). We consider the belt to be a prestressed shear deformable beam. The rigid pulley is a cylinder rotating with a constant velocity $\omega$. The present work is an extension of the previously published paper [6], where the similar problem was discussed for a flat pulley. The introduction of the (small) transverse displacements and of the corresponding force factors is the novel point in this research. The belt is assumed prestressed, which also differsentiates the present model from [6]. In this work, we consider quasistatic motion, because the inertial terms do not qualitatively change the behavior in the belt–pulley contact area.

In recent related works on the topic (see [2, 8]), we find similar formulations with large displacements and rotations. However, the authors of [8] use some simplifying assumptions in the contact segment. The friction interaction is assumed to occur at the belt’s middle axis, whereas in the papers [2, 6] it is at the bottom fibers. Shifting the contact forces to the belt’s middle axis leads to certain simplifications in the problem formulation, for instance, to constant stretch in the sticking region. The simplifications are very helpful, because the problem at hand is exceptionally stiff and numerically unstable. The reason for these difficulties is that the contact forces are particularly concentrated at the boundaries of the contact region (see, e.g., [1]). We investigate two models in this note: the one with regular sticking and sliding segments and the one with a regular sticking segment and the concentrated contact interaction instead of a sliding segment. We compare the results of the first model with the results given in [8].

The present note is intended to find out how it affects the friction force if we set the friction interaction at the belt middle axis. To do this, we discuss a more complicated friction interaction model than those treated in [8], namely, not shifting the line of action of friction forces to the belt middle line, and demonstrate possible erroneous effects.

## Problem formulation and model with slip segment

### Problem formulation

Let us start from the incremental equations for a Cosserat rod. These linearized equations may be derived by varying the equations of the geometrically nonlinear Cosserat theory; for details, see [3, Sect. 7.2] (in Russian) or [9, Sect. 3.1.2] (in English). They are written as1Here, $\tilde{\mathbf {Q}}$, $\tilde{\mathbf {M}}$, $\tilde{\mathbf {q}}$, $\tilde{\mathbf {m}}$, $\tilde{\varvec{\theta }}$, and $\mathbf {u}$ describe the increments: force, moment, external distributed load and moment, angle, and displacement. The quantities obtained from the solution of the nonlinear problem are the force $\mathbf {Q}$, the moment $\mathbf {M}$, and the position vector $\mathbf {r}$. The remaining values in Eq. (1) are the compliance tensors of torsion bending and tension shear $\mathbf {A}$ and $\mathbf {B}$ (the tensor of coupling terms is set to 0). The linear constitutive equations (elasticity relations) are implied in the last two equations of (1). Note that a prime denotes the derivative with respect to the material coordinate *s*, which is the arc coordinate in the undeformed configuration.

In the current problem, the prestressed configuration is simply the axially tensed straight rod: the moment vanishes, $\mathbf {M}$ = 0, and the force and position vector derivative are constant and directed along the rod axis:2In these relations, we have introduced the tension stiffness $b_1$, which is the inverse of the tension compliance, the component of $\mathbf {B}$, the axial strain $\varepsilon _0$, and the unit vector of the horizontal axis $\mathbf {i}$. We have utilized the Biot strain measure and the linear elasticity relations in these formulas. With these simplifications and the assumption of plane deformation, we may rewrite the balance equations in Eq. (1) as follows

3 $$\begin{aligned} T' + t = 0, \quad Q' + q = 0, \quad M' - b_1 \varepsilon _0 u_y' + (1+ \varepsilon _0) Q + \frac{h}{2}t = 0. \end{aligned}$$

Here, we have renamed the components of force and moment increments: $\tilde{\mathbf {Q}} = (T-b_1 \varepsilon _0)\mathbf {i} + Q \mathbf {j}$, $\tilde{\mathbf {q}} = t \mathbf {i} + q \mathbf {j}$, and $\tilde{\mathbf {M}}= M \mathbf {k}$. The unit vector $\mathbf {j}$ is oriented vertically. We have used the relation $m = th/2$ for the external moment load of the friction force *t* acting on the lower fibers of the belt with the thickness *h*. The transverse force *q* and the friction force *t* are yet unknown in the contact area.

The elasticity relations follow from the last two equations in Eq. (1),

4 $$\begin{aligned} M = a\theta ', \quad Q = b_1 \varepsilon _0 \theta + b_2 (u_y' - (1 + \varepsilon _0) \theta ), \quad T = b_1 \varepsilon + b_1 \varepsilon _0, \end{aligned}$$

where we skip the tilde above the angle and introduce the shear stiffness $b_2$ and the bending stiffness *a*. Along with the axial strain $\varepsilon = u'_x$, the belt’s kinematics is described by the rotation angle $\theta$ (with the corresponding bending strain $\kappa = \theta '$) and by the shear strain $\gamma =u'_y- (1+\varepsilon _0)\theta$ with the initial strain $\varepsilon _0$. There exists the widely accepted second-order theory of beams (see, e.g., [7, p. 87]), which is not used in this paper; however, we checked that the differences in results are so small that they cannot be seen if plotted in the graphs below.

**Fig. 1:**
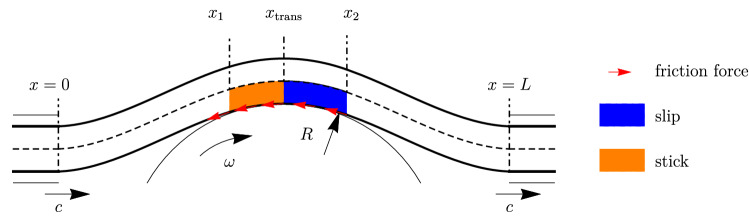
Belt drive as an axially moving beam in frictional contact with pulley as a rigid cylinder

We show the calculation scheme in Fig. 1. The belt is modeled as a shearable beam, and the pulley is a rigid body. The belt enters the studied domain through the bearing with the spatial coordinate $x=0$ and leaves it through the bearing with the coordinate $x=L$. Material with the length *c* enters and leaves the domain per unit time at the places of the inlet and outlet. The vertical coordinates of the inlet and outlet are zero. The velocity of contacting points of the rigid pulley $v=\omega R$ is slightly different from *c*. We consider the steady-state mode: at each point of space, the velocity and strain are constant in time.

The entire belt under consideration may be divided into three regions with the contact segment in the middle ($x_1,x_2$) between two free segments (spans) (see Fig. 1). The contact segment boundaries are not a priori prescribed.

### Contact segment

We assume that there are no gaps between the pulley and belt in the contact segment (full contact). The contact with the pulley (a rigid body) determines the vertical component $u_y$ of the translation; in the current case with small strains superposed upon the prestressed configuration, it can be described by the parabola

5 $$\begin{aligned} u_y = \delta - R + \sqrt{R^2 - (1+\varepsilon _0)(x-L/2)^2} = \delta - (1+\varepsilon _0)\frac{(x-L/2)^2}{2R} + \ldots . \end{aligned}$$

Here, $\delta$ is the pulley vertical displacement. In the following, we restrict ourselves within quadratic approximation. We differentiate this equation and substitute the formula for $u_y'$ in the constitutive equation for *Q* from Eq. (4):

6 $$\begin{aligned} Q = \frac{b_2}{2R}(L-2x)(1+\varepsilon _0) + (b_1\varepsilon _0 - b_2 (1 + \varepsilon _0))\theta = Q(\theta ,x). \end{aligned}$$

This equation may be used to determine the normal pressure from Eq. (3):

7 $$\begin{aligned} q = \frac{b_2}{R}(1+\varepsilon _0) + (b_2 - \varepsilon _0(b_1-b_2))\theta '. \end{aligned}$$

First, we consider the stick behavior in the contact segment, which results in the prescribed constant bottom velocity and extension. In the contact segment, between the yet unknown points $x_1$ and $x_2$, the lower fibers of the belt have the strain (cf. [4, Eq. (21)] or [6, Eq. (2)])

8 $$\begin{aligned} \varepsilon _{\mathrm {bottom}} = \frac{\omega R}{c} - 1. \end{aligned}$$

The beam kinematics admits the following representation of the strain:

9 $$\begin{aligned} \varepsilon _{\mathrm {bottom}} = \varepsilon +\varepsilon _0 + \frac{h}{2} \theta ', \end{aligned}$$

where *h*/2 is the half-height of the beam and $\varepsilon$ is for the strain at the belt middle axis.

The constancy of the bottom strain $\varepsilon _{\mathrm {bottom}}$ in the contact segment leads to the following equation:

10 $$\begin{aligned} \varepsilon '_\mathrm {stick}= -\frac{h}{2} \theta _\mathrm {stick} ''. \end{aligned}$$

Using this equation, the constitutive Eq. (4) and the force balance Eq. (3), we derive the friction force in the sticking segment

11 $$\begin{aligned} t_\mathrm {stick} = \frac{b_1 h}{2} \theta _\mathrm {stick}'' . \end{aligned}$$

Now, we can substitute Eqs. (6) and (11) in the balance of moments and derive the second-order differential equation for the angle12or, in shorter form,

13 $$\begin{aligned} \theta _\mathrm {stick}'' - \alpha _\mathrm {stick}^2 \theta _\mathrm {stick} = \alpha _\mathrm {stick}^2 \frac{x-L/2}{R} , \end{aligned}$$

where

$$\begin{aligned} \alpha _\mathrm {stick} = \sqrt{\frac{4\beta }{4a + b_1h^2}}. \end{aligned}$$

We integrate Eq. (13) and write the angle $\theta$ as a function of *x* and two integration constants,

14 $$\begin{aligned} \theta _\mathrm {stick} = C_1 \cos {(\alpha _\mathrm {stick} x)} + C_2 \sin {(-\alpha _\mathrm {stick} x)} - \frac{x-L/2}{R}. \end{aligned}$$

The axial strain $\varepsilon$, the friction force *t*, and the normal pressure *q* depend on the angle of rotation as given in Eqs. (7), (8), (9), and (11). Afterwards, the moment *M*, the tension force *T*, and the transverse force *Q* in the contact segment may be obtained from the constitutive equation (4).

### Free segments

Let us proceed to the free segments. The constitutive equation (4) holds in the free segments.

The external loads are zero: $m=0$, $q = 0$, and $t = 0$, which leads to the following simplified balance equations:

15 $$\begin{aligned} T'= 0, \quad Q' = 0, \quad M' -b_1 \varepsilon _0 u_y' + (1+\varepsilon _0) Q = 0. \end{aligned}$$

The first two equations may be integrated with the unknown integration constants resulting in the expressions (they are unequal in the left and right free spans in the working belt drive)16where we have used the corresponding subscripts for the left and right free segments.

We may derive the derivatives of the transverse displacement from Eq. (16) as a function of the angle and transverse force,17Now, we substitute this equation in the balance of moments and arrive at the second-order differential equation for the angle18We can integrate this equation and represent the solution in the form19where we have used the corresponding combinations of the constant $\varepsilon _0$ and stiffness factors in $\alpha _0$ and $\alpha _1$ (not provided here). By $C_i,i=3,4,5,6$, we denote the integration constants. To eliminate unnecessary constants, we can directly use the trivial boundary conditions: $\theta _\mathrm {left}(0) = 0$ and $\theta _\mathrm {right}(L)=0$ caused by the geometry of the problem (see Fig. 1). It is easy to write the derivative of the angle $\theta$ and, using the constitutive equation and the constancy of the transverse force *Q*, the moment *M* in the free segment.

With the known angle, we can now integrate the equation for the transverse displacement, Eq. (17). One integration constant arises in each segment, and we use the boundary conditions $u_y=0$ at $x=0,L$ to determine them.

Thus, the entire solution in the left (right) free segment depends on three unknown integration constants: $T_\mathrm {left}$ ($T_\mathrm {right}$), $Q_\mathrm {left}$ ($Q_\mathrm {right}$), and $C_4$ ($C_6$).

### Solution with slip

In this subsection, we derive the governing equations relevant in the slip segment which is located near the point $x_2$ where the belt leaves the pulley (see Fig. 1).

Because the transverse displacement is known under the full contact assumption (see Eq. (5)), we may again derive the transverse force *Q* and the normal reaction *q* as in the stick region above, Eqs. (6) and (7). In the slip region, the Coulomb law with the friction coefficient $\mu$ couples the axial and normal components of external load; therefore, the friction force becomes

20 $$\begin{aligned} t_\mathrm {slip}=\mu q_\mathrm {slip} = \mu \left( (b_2- \varepsilon _0(b_1-b_2))\theta _\mathrm {slip}' + \frac{b_2}{R}(1+\varepsilon _0) \right) . \end{aligned}$$

Now, we can substitute it and the constitutive equation into the balance of moments and derive the second-order differential equation for the angle21This equation can be integrated with appearance of two integration constants, $C_7$ and $C_8$,

22 $$\begin{aligned} \theta _\mathrm {slip} = C_7 \exp {(-x(\alpha _\mathrm {slip1} + \alpha _\mathrm {slip2}))}+ C_8 \exp {(x(\alpha _\mathrm {slip1} - \alpha _\mathrm {slip2}))} +\alpha _\mathrm {slip0}, \end{aligned}$$

where $\alpha _\mathrm {slip0}$, $\alpha _\mathrm {slip1}$ and $\alpha _\mathrm {slip2}$ are combinations of the constant problem parameters used for simplicity’s sake.

We substitute the obtained angle in the expressions for the friction force, normal reaction, moment, and transverse force. Having obtained the friction force, we proceed to the balance equation for the tension, Eq. (3). This equation admits integration with the integration constant $T_\mathrm {slip0}$. We obtain $\varepsilon _\mathrm {slip}$ from the constitutive equation.

Now, we proceed to the formulation of boundary conditions. The angle and the transverse displacements are zero at the ends $x=0,L$ of the considered part of the belt,

23 $$\begin{aligned} x = 0: \quad u_y=0,\;\theta _\mathrm {left} = 0; \quad x = L: \quad u_y=0,\; \theta _\mathrm {right} = 0. \end{aligned}$$

These boundary conditions are used to eliminate the integration constants in $\theta _\mathrm {left}$, $\theta _\mathrm {right}$, $u_{y,\mathrm {left}}$, and $u_{y,\mathrm {right}}$.

At the boundary points of the contact segment $x_1$ and $x_2$, we impose the continuity of the transverse displacement, angle, tension, transverse force, and moment.24At the transition point between the sliding and sticking regions, we may postulate that the transverse displacement, the angle, the tension, the transverse force, and the moment suffer no jumps. However, the transverse displacement $u_y$ is prescribed by the pulley displacement (the same formula (5) in both sticking and sliding regions). Consequently, the continuity of transverse force is nothing else as the continuity of angle, which follows from constitutive Eq. (4). Hence, there remain 3 boundary conditions at the transition point $x_\text {trans}$:25The last condition is the conservation of material length (see, e.g., [6]). According to this condition, the total integral of $\varepsilon$ over the considered segment (0, *L*) must vanish:

26 $$\begin{aligned} \int \limits _0^{L}{\varepsilon \mathrm{d}x}=0. \end{aligned}$$

There are altogether 14 equations for the 14 unknown values listed below:

- $C_1$ and $C_2$ in the stick region, Eq. (14);
- $C_7$ and $C_8$ in the slip segment, Eq. (22);
- $x_1$ and $x_2$, which are the boundaries of the contact segment;
- $x_\mathrm {trans}$, which is the transition point between the stick and slip segment; and
- $Q_\mathrm {left}$, $T_\mathrm {left}$, and $C_4$ in the left free segment and $Q_\mathrm {right}$, $T_\mathrm {right}$, and $C_6$ in the right segment, and $T_\mathrm {slip0}$ in the slip segment.

The conditions form a nonlinear algebraic problem, which may be solved by the Newton method, e.g., implemented in the built-in function FindRoot of Wolfram Mathematica. The problem under consideration appears to be numerically unstable when we vary its parameters, including the initial guesses. Nevertheless, we succeed in solving it in some special cases. From a numerical point of view, it is helpful to prescribe the parameter $x_\mathrm {trans}$ (the coordinate of the transition point between the stick and slip segments) and release the angular velocity $\omega$ of the pulley (i.e., to treat $\omega$ as an unknown parameter). This kind of replacement may also be found in [8].

**Fig. 2:**
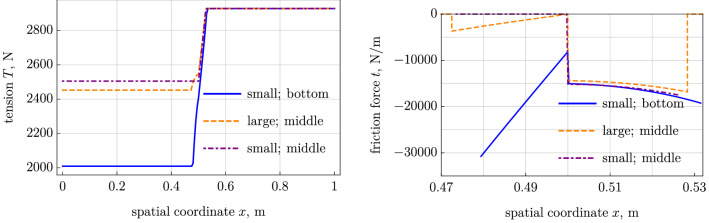
Small-strain model with bottom and middle-axis contact interaction vs large-strain model with middle-axis contact interaction [8]: (*left*) tension and (*right*) friction force.

**Fig. 3:**
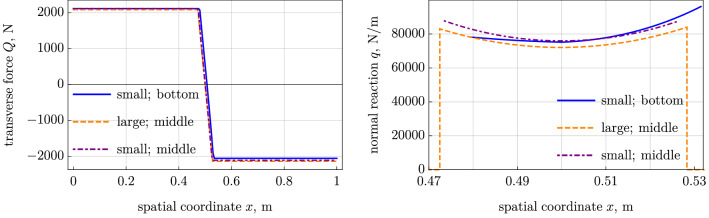
Small-strain model with bottom and middle-axis contact interaction vs large-strain model with middle-axis contact interaction [8]: (*left*) transverse force and (*right*) normal reaction

**Fig. 4:**
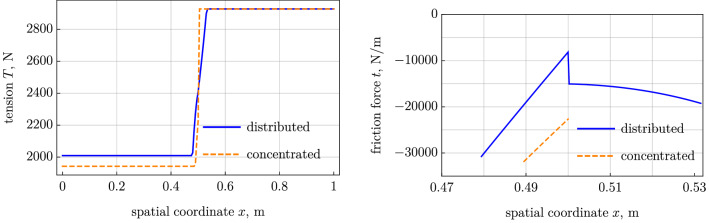
Model with distributed sliding segment vs model with concentrated reaction: (*left*) tension and (*right*) friction force

**Fig. 5:**
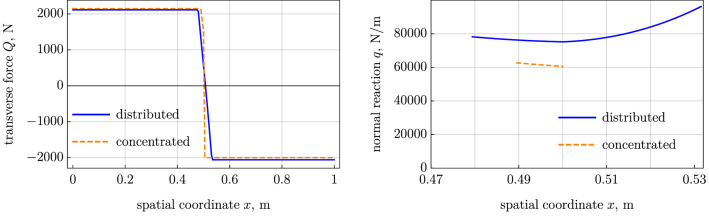
Model with distributed sliding segment vs model with concentrated reaction: (*left*) transverse force and (*right*) normal reaction

The results are plotted in Figs. 2, 3, 4, and 5 compared with another cases. The initial stress in this case is set to $\varepsilon _0=0.00493$ (cf. $\varepsilon _0=0.004873$ in the case with lumped force) in order to equalize the tension at the right point $x=L$. We also show the results of the small-strain model with the friction forces shifted to the belt middle line (with $\varepsilon _0=0.005421$). It seems to be useful, because the large-strain and small-strain models have significant differences apart from the action line. The results are easy to obtain with the technique given above with $h=0$ in the kinematic relation for the axial strain, Eq. (9), and in the balance of moments, Eq. (3), where the moment load *m* vanishes.

### Numerical results and comparison

The numerical parameters are the following: $x_2=0.5$, $L=1$ is the length of the considered part of the belt (SI units throughout the paper), $E=5\cdot 10^7$ is the Young’s modulus, $\nu =0.45$ is the Poisson ratio, $h=0.1$ is the height of the squared cross-section of the belt, $c=1$ is the length of the belt material that enters and leaves the domain at the inlet and outlet, $k=1.1$ is the shear coefficient, $R=0.7$ is the radius of the pulley, and $\mu =-0.2$ is the friction coefficient for the Coulomb law used in the next subsection (with negative sign we describe the case of the driven pulley which brakes the belt motion). We use the stiffness formulas: $a=Eh^4/12$, $b_1=E h^2$, and $b_2=kEh^2/2(1+\nu )$. The displacement of the pulley is set at $\delta =0.05$. To compare the results obtained in this paper with the results of work [8], we prescribe the strain of the initial configuration $\varepsilon _0$ so that the tension in the right point $x=L$ is equal in both models.

It is interesting to compare the results of our quasistatic model with slip with the results of the geometrically nonlinear model developed in [8]. The contact interaction is characterized differently in that model: It is assumed that the friction force acts at the belt middle axis; therefore, the height of the belt *h* equals zero in the kinematic relation for the axial strain, Eq. (9), and in the balance of moments, Eq. (3), where $m=0$.

The most evident differences in the results are seen in Fig. 2 in the axial force and in the friction force in the sticking segment (note that in the small-strain model we adjusted the initial strain $\varepsilon _0$ to equate the tension forces of two models at the right end of the belt). A large part of the friction interaction is lost when we assume that it occurs at the belt middle axis. This difference cannot be explained by the large strains and rotations, because we have chosen a moderate pulley displacement $\delta$ and a moderate initial strain $\varepsilon _0$. The small-strain model with the contact interaction shifted to the middle line shows the results similar to that of the large-strain model; only difference is the vanishing friction force in the sticking region.

The contact segment is slightly shorter in the model with small strains and bottom contact interaction, because it enables larger friction forces in the sticking segment. This insignificantly affects the transverse force, which is presented in Fig. 3.

## Solution with concentrated slip region

In this section, we study the case with a slip region concentrated in the point where the belt leaves the pulley. To introduce this model, we need to talk about the jump conditions at this point. The governing equations in the segments remain the same, and we do not repeat them. Let us consider the boundary conditions, including the matching and jump conditions.

At the boundaries of the considered belt, we prescribe the following angles and axial displacements:

27 $$\begin{aligned} x = 0: \quad \theta _\mathrm {left} = 0, \; u_{y,\mathrm {left}}= 0;\quad x = L: \quad \theta _\mathrm {right} = 0, \; u_{y,\mathrm {right}}=0. \end{aligned}$$

These equations have been already used in elimination of integration constants (see Sect. 2.3).

We can also write four kinematic matching conditions at the boundaries of the contact segment:28Because we anticipate no concentrated forces or moments in the point $x_1$, where the belt enters the pulley, owing to adhesion (as usually done in the literature on belt drives), we may write

29 $$\begin{aligned} x_1: \quad M_\mathrm {left} = M_\mathrm {stick}, \quad T_\mathrm {left} = T_\mathrm {stick}, \quad Q_{\mathrm {left}} = Q_{\mathrm {stick}}. \end{aligned}$$

We assume that there is a concentrated normal reaction $F_y$ at the point $x_2$, where the belt leaves the pulley. This concentrated contact interaction may be regarded as the limit case of an extremely narrow sliding segment. This assumption involves the jump conditions in the point $x_2$.30where, due to the Coulomb law, the axial and normal components are related by the friction coefficient $\mu$ whose sign depends on the direction of relative motion. We have also used the Coulomb law in studying the sliding behavior in the regular sliding segment above. See consideration of jump conditions in the belt drive mechanics in [4] and general treatment of the jump conditions in [5]. The jump conditions were also applied in [6] to the steady-state problem with contact of a traveling beam with a flat plate.

The conservation of the material length *L*, Eq. (26), now without the sliding region, can again be utilized.

We have altogether 11 conditions for the 11 unknowns listed below:

- $C_1$ and $C_2$ in the stick region, Eq. (14);
- $x_1$ and $x_2$, which are the boundaries of the contact segment;
- $Q_\mathrm {left}$, $T_\mathrm {left}$, and $C_4$ in the left free segment and $Q_\mathrm {right}$, $T_\mathrm {right}$, and $C_6$ in the right segment; and
- $F_y$, the unknown lumped force in the point $x_2$ where the belt leaves the pulley.

The conditions form a nonlinear algebraic problem solved by the Newton method. As above in the case with slip, it is advantageous from the numerical point of view to treat the angular velocity $\omega$ as an unknown variable and the coordinate $x_2$ as a prescribed parameter. We plot the case with the sliding segment and the case with the concentrated contact reaction (see Figs. 4 and 5). The contact area is significantly narrower in the case without sliding friction, because the most part of the contact interaction can be associated with the concentrated forces, $F_y=3502$ N and $F_x=\mu F_y = -700.4$ N.

## Conclusions

In the framework of prestressed belt model with small strains, but with nonlinear contact with the pulley, we could show that shifting the line of action of friction forces to the belt middle axis results in particular losses in the calculated friction force. This may and should be taken into account in further investigation of the belt drive behavior. The current model can be extended to two-pulley and multi-pulley configurations and should be helpful to obtain an initial guess for future studies treating the belt as a beam with large deformations.
